# Advances in the Effects of Heat Stress on Ovarian Granulosa Cells: Unveiling Novel Ferroptosis Pathways

**DOI:** 10.3390/vetsci11100464

**Published:** 2024-10-01

**Authors:** Zhen Zhu, Jiang Wu, Yuguo Wen, Xiaocheng Wu, Huimingda Bao, Min Wang, Kai Kang

**Affiliations:** 1 Department of Animal Science, College of Coastal Agricultural Sciences, Guangdong Ocean University, Zhanjiang 524088, China; 202111331236@stu.gdou.edu.cn (Z.Z.); wuj@gdou.edu.cn (J.W.);; 2 Department of Veterinary Medicine, College of Coastal Agricultural Sciences, Guangdong Ocean University, Zhanjiang 524088, China

**Keywords:** heat stress, ovarian granulosa cells, hormone, apoptosis, ferroptosis, mechanism

## Abstract

**Simple Summary:**

Heat stress exerts extensive and intricate effects on germ cells, attracting significant attention from researchers. This article provides a comprehensive review and summary of the impacts and mechanisms of heat stress on ovarian granulosa cells. The systematic evaluation of heat stress effects on ovarian granulosa cells encompasses alterations in steroid hormone levels, oxidative stress, apoptosis, and mitochondrial function, with detailed discussions on several key mechanisms. Furthermore, this study analyzes the mechanism and potential relevance of ferroptosis in ovarian granulosa cells under heat stress conditions. Looking ahead to the future, gaining a deeper understanding of this complex mechanism will serve as a theoretical foundation for exploring possible interactions between heat stress and ferroptosis.

**Abstract:**

Heat stress has been one of the key research areas for researchers due to the wide-ranging effects and complex mechanisms of action of its stress product reactive oxygen species (ROS). The aim of this paper is to comprehensively review and summarize the effects of heat stress on ovarian granulosa cells and their mechanism of action. We systematically reviewed the effects of heat stress on ovarian granulosa cells, including intracellular steroid hormone changes, oxidative stress, apoptosis, and mitochondrial function. Meanwhile, this paper discusses in detail several major mechanisms by which heat stress induces apoptosis in ovarian granulosa cells, such as through the activation of apoptosis-related genes, induction of endoplasmic reticulum stress, and the mitochondrial pathway. In addition, we analyzed the mechanism of ferroptosis in ovarian granulosa cells under heat stress conditions, summarized the potential association between heat stress and ferroptosis in light of the existing literature, and explored the key factors in the mechanism of action of heat stress, such as the signaling pathways of Nrf2/Keap1, HSPs, and JNK, and analyzed their possible roles in the process of ferroptosis. Finally, this paper provides an outlook on the future research direction, describing the possible interaction between heat stress and ferroptosis, with a view to providing a theoretical basis for further understanding and revealing the complex mechanism of ferroptosis occurrence in ovarian granulosa cells under heat stress.

## 1. Introduction

Ovarian granulosa cells (GCs), named for the granules contained in their cytoplasm, are the major functional cells in the animal ovary, closely associated with follicular development and the primary site of the follicular secretion of steroid hormones such as estradiol and progesterone. Researchers have found that only 1% of follicles can reach the pre-ovulation stage, and the remaining 99% will undergo atrophic degeneration. A variety of factors that are essential for follicle growth and reproduction, including steroid hormones, growth factors, and cytokines, are secreted by granulosa cells [1,2]. Follicular communication is also achieved through gap junctions in the form of transverse zonular protrusions (TZPs) originating from GCs [3], suggesting that GCs also regulate follicular atresia. In addition, during the LH surge prior to follicular ovulation, the oocyte obtains more than 85% of its metabolic requirements through gap junctions with the granulosa cells, which also provide the appropriate pH for its growth, demonstrating that the granulosa cells also play an important nutritional role [3].

The decline in animal reproductive performance in hot climates is well documented, and inevitable global warming and intensive farming to achieve high yields are predicted to greatly limit it [4]. Among the various forms of stress, heat stress has been identified as a key factor affecting reproductive health, particularly in areas prone to high environmental temperatures. Heat stress can directly interfere with thermoregulation and lead to systemic effects, including alterations in endocrine function and follicular development, particularly affecting granulosa cells [5]. In addition, climate change has led to an increase in global temperatures, further exacerbating the relevance of heat stress in animal husbandry, where reproductive performance, which is closely linked to the granulosa cell, is critical to livestock production. In thermoregulation, an isothermal zone refers to the temperature range within which an organism can maintain a relatively constant body temperature by adjusting metabolic activity and heat production. This zone is defined by an upper critical temperature (UCT) and a lower critical temperature (LCT). If the environmental temperature exceeds the UCT for a prolonged period, or if the organism’s ability to dissipate heat becomes insufficient to counterbalance the heat gained, heat stress will occur, potentially compromising the body’s ability to regulate its internal temperature. The mechanisms by which heat stress affects GCs are complex and diverse. These include the induction of apoptosis by affecting steroid levels [6], mitochondrial damage [7], endoplasmic reticulum stress [8], changes in the expression of heat shock proteins (HSPs) [9], and the activation of the Fas cell surface death receptor/FAS ligand (FAS/FASL) extrinsic apoptotic pathway [10]. However, the most critical mechanism is the excessive production of reactive oxygen species (ROS), which leads to oxidative stress. Excessive reactive oxygen species are involved in the HS-induced apoptotic pathway as a key influencing factor, such as mitochondrial damage, endoplasmic reticulum stress, and FAS/FASL. Ferroptosis represents a novel form of cell death that differs from traditional forms such as apoptosis, necrosis, and autophagy. It is a non-apoptotic cell death dependent on ferroptosis, triggered by decreased glutathione peroxidase 4 (GPX4) activity, intracellular iron accumulation, and lipid peroxidation. The core mechanisms of ferroptosis include an imbalance between iron-dependent lipid peroxidation and the antioxidant system. Recent studies have indicated that heat stress may contribute to an increase in intracellular free iron levels by disrupting the equilibrium of intracellular iron metabolism [11]. In addition, heat stress may inhibit the function of the antioxidant system, such as decreasing the activity of GPX4, further exacerbating oxidative stress in cells, and inducing ferroptosis [12]. These findings show that when ovarian granulosa cells are exposed to heat stress, the ferritin deposition pathway is also activated. This leads to a decrease in animal fertility and affects production performance.

Therefore, this review will present a comprehensive summary of the mechanisms involved in heat stress-induced apoptosis and ferroptosis in ovarian granulosa cells, with a particular focus on the roles of steroid hormone production, oxidative stress, mitochondrial function, endoplasmic reticulum stress, the expression of heat shock proteins, and the extrinsic apoptotic pathway of Fas. The objective is to provide scientific evidence to maintain and improve the health and reproductive function of animals, with a view to understanding the mechanisms of injury to GCs and the possible mechanisms of ferroptosis, as well as the cross-pathways in heat stress conditions.

## 2. Heat Stress Affects Steroid Hormone Production

Granulosa cells are vital for the growth and maturation of oocytes in the majority of animal species, including mammals and birds (Table 1). This is due to their role in the synthesis and secretion of steroid hormones, such as estradiol (E2), progesterone (P4), and follicle-stimulating hormone (FSH) [13]. Nevertheless, numerous studies on mammals and avian animals have demonstrated that elevated ambient heat stress levels prevent the biosynthesis of steroid hormones in their glucocorticoid cells, leading to a reduction in the lifespan of these cells.

### 2.1. On Estradiol

Compared with mammals, birds such as chickens show an excessive increase in estradiol levels at high temperatures. This temporary increase has not been observed in other mammals. This may be due to their special compensatory mechanisms. However, in terms of overall changes in levels, the estradiol synthesis levels of both birds and mammals decrease after heat stress.

#### 2.1.1. In Mammals

In rodents and artiodactyls, heat stress causes a decrease in estradiol levels by inhibiting aromatase activity and reducing the expression of genes involved in the production of key steroid hormones. However, in rodents, heat stress is also closely associated with an increase in prolactin and glucocorticoids.

##### In Artiodactyla

Heat stress (HS) is classified into acute and chronic categories. When cows are subjected to chronic HS, it is more probable that lower E2 levels will be observed in the follicular fluid compared to acute HS [14,22]. It is hypothesized that reduced estradiol levels are responsible for impaired granulosa cell function and even apoptosis under conditions of heat stress. This is evidenced by a higher incidence of synchronized estrus and natural estrus ovulation failure in cows [44]. This resulted in the discovery that the mRNA expression of CYP11A1, CYP19A1, and FSHR was markedly elevated in granulosa cells from cows treated with Insulin-like growth factor 1 (IGF-1). It has been established that IGF-1 independently affects the production of granulocyte steroids, or acts in conjunction with FSH, and does not affect the synthesis of estradiol [15]. Furthermore, heat stress has been demonstrated to result in a reduction in steroidogenic gene mRNA expression and estrogen synthesis via the BAX-BCL2 pathway [16]. This provides compelling evidence for the existence of an estradiol-regulated pathway. In addition, heat stress can reduce the level of estrogen 2 (E2) in cow follicular fluid by eliminating the aromatase activity of cow granulocytes [17,18]. One of the earlier studies indicated that heat stress may be seasonal, with a reduction in androstenedione production in cow GCs occurring during the cooler autumn months. This suggests that summer heat may have a conductive effect on follicular function [18]. Subsequently, Roth et al. reported a delayed effect of heat stress on the coercive effects of heat stress on granulosa cells of pre-ovulatory follicles in dairy cows. Different patterns can be observed in the granulosa cells of each follicle: the granulosa cells of medium-sized follicles produce less estradiol, while the granulosa cells of pre-ovulatory follicles produce less active estradiol [14]. In addition, messenger RNA (mRNA) expression of the nuclear receptor steroidogenic factor (SF-1) is considered to be the main regulatory factor of steroidogenesis. After heat treatment, its expression is reduced, and inhibition of the mRNA expression of key genes related to steroidogenesis (such as STAR, CYPA11A1, etc.) can be observed in dairy cows [16]. This is consistent with the changes in the expression of key genes for steroid hormone synthesis, such as P4 and E2, that were inhibited by heat stress in dairy cows, as reported by Khan et al. [19], suggesting that steroid hormone biosynthesis is inhibited in ovarian granulosa cells.

The steroidogenic activity and viability of Egyptian buffalo GCs were not affected when they were cultured under heat stress conditions for an extended period of time. This was evidenced by an increase in E2 concentration in the cell culture medium and the observation that high-temperature treatment did not affect the secretory capacity of the GCs when cultured in the presence of added serum, which mimicked normal physiological conditions [14,20]. Similarly, the follicular fluid composition of dominant follicles is not affected by acute hyperthermia [21]. It can be assumed that heat stress reduces the release of adrenocorticotropic hormone, which may explain the contradictory results observed in animal and cellular experiments [20,24]. Similar studies have shown that buffalo granulosa cells secrete large amounts of IGF-1 within 48 h after the onset of heat stress, and that the concentration of IGF-1 decreases within 48–72 h, with E2 showing a similar trend. This indicates that buffalo granulosa cells are resistant to the disorder of GC steroidogenesis caused by heat stress, which may be due to the anti-apoptotic effect of IGF-1 [20]. This may explain the discrepancy between the findings of the study by De Castro e Paula and those of Roth et al., in which chronic heat stress was found to result in lower estradiol levels in the follicular fluid of dairy cows, while acute heat stress did not affect estradiol [14,22].

In most species of even-toed ungulates, including pigs, heat stress affects ovarian granulosa cells in a similar way, and it has been shown that IGF-1, leptin, and FSH are involved in the proliferative, apoptotic, and secretory activities of sow ovarian cells, promoting CYP11A1 expression and preventing the effects of stress on ovarian cell function [24]. Heat stress has also been shown experimentally to affect E2 biosynthesis in porcine GCs by increasing heat shock protein 70 (HSP70) and indirectly inhibiting Smad3 phosphorylation and nuclear translocation [25].

##### In Rodents

As with even-toed ungulates, high-temperature environments have been shown to inhibit the mRNA expression of the key steroid hormone production genes STAR, CYP11A1, and CYP19A1 [29]. Heat stress also inhibits aromatase activity in granulosa cells, resulting in a low follicular capacity to produce estradiol [2]. This inhibition of aromatase may be due to targeted inhibition of prolactin and glucocorticoid hormones caused by heat-induced increases in their levels [2]. The effects of reduced estradiol levels, which are regulated by various pathways and mechanisms, on granulosa cells are evident. In E2-treated mice, there is an increased expression of the nucleic acid endonuclease Ape1 in the cerebral cortex, indicating that E2 plays a role in neuroprotection by enhancing the expression of oxidative stress-responsive proteins and reducing oxidative DNA damage. This suggests that heat stress may decrease the secretion of E2, thereby impairing its ability to promote the activation of the oxidative stress-responsive protein, purine endonuclease (Ape1) [30]. Reducing the expression of this multifunctional protein, which is involved in DNA repair and redox regulation, increases the likelihood of oxidative DNA damage inducing apoptosis in granulosa cells [30]. HSP70 is not entirely beneficial. It has been reported that when heat stress occurs, HSP70 not only protects cells, but also hinders the production of hormone-sensitive steroids in rat corpus luteum cells [31].

In addition, heat stress can induce estrous cycle disorders in female rats, leading to ovarian hyporesponsiveness or ovarian dysfunction. This is due to the restricted levels of steroid hormones, resulting in lower levels of luteinizing hormone (LH), which in turn suppresses insulin (INS) secretion. This suppression reduces the body’s glucose metabolism and causes disruptions in the hypothalamic–pituitary–gonadal (HPG) axis, thereby impairing the feedback regulation of the hypothalamic–pituitary–ovarian (HPO) axis [32].

#### 2.1.2. In Poultry

In response to elevated temperatures, egg-laying hens demonstrate a decline in egg production and a reduction in ovarian weight, accompanied by an increase in reactive oxygen species (ROS) levels [38]. However, no significant changes in body temperature were observed in the experimental subjects [38]. Some experimental evidence indicates that under HS conditions, laying hens activate the hypothalamic–pituitary–adrenal (HPA) axis, resulting in elevated levels of corticotropin-releasing hormone (CRH) and corticosterone in the blood and follicular fluid. This activation stimulates the Fas and TNF-α signaling pathways, thereby increasing oxidative stress [45]. The results indicate that the adverse effects of heat stress on the organism are primarily mediated by its influence on hormonal levels. In addition, the E2 levels in the pre-ovulatory granulosa cells of chickens treated at 43 °C continuously increased after 12 h of treatment, exceeding the control group after 24 h, and then gradually decreased. In contrast, in the 41 °C treatment group, the E2 levels initially exhibited a comparable profile to the control group, but subsequently demonstrated a continuous downward trajectory, reaching a level below that of the control group after only 36 h of treatment [41]. This transient increase in E2 levels at 43 °C might be due to a compensatory effect unique to chicken granulosa cells, a phenomenon not yet reported in other animals. The underlying mechanism remains to be explored.

In the context of laying hens, steroid hormones such as estradiol (E2) and progesterone (P4) are produced by granulosa cells and theca cells. From a transcriptomic perspective, the deficiency of solute carrier family 5 member 5 (SLC5A5) has been observed to inhibit the proliferation of chicken ovarian germ cells, the synthesis and secretion of steroid hormones, and to promote apoptosis. It is proposed that SLC5A5 exerts its effects by regulating the cellular uptake of anions, including iodine, thiocyanate, and perchlorate [39]. Similar to mammals, studies have observed that heat stress reduces the expression of CYP19A1 in both chickens and ducks. This suggests that heat stress lowers E2 levels in granulosa cells by inhibiting the pathway responsible for E2 production [40,41].

### 2.2. On Progesterone

In both mammals and birds, the expression of genes such as CYP11A1 and StAR is affected by heat stress and P4 synthesis is inhibited. However, when birds respond to heat stress, the expression levels of some genes show time-dependent changes and there are also some differences between different species (such as chickens and ducks). This indicates that the response mechanisms of different species to heat stress are different.

#### 2.2.1. In Mammals

The effects of heat stress on P4 synthesis in the granulosa cells of artiodactyls and mammals are different. Although heat stress inhibits the expression of CYP11A1 and StAR genes, the P4 level in granulosa cells of artiodactyls usually does not change significantly. This may be due to other sources of P4 compensating for this loss. In contrast, P4 synthesis in the granulosa cells of rodents is significantly reduced.

##### In Artiodactyla

Compared to the significant changes observed in E2 levels, the progesterone levels in granulosa cells show no significant changes even when the mRNA expression of two key regulatory genes, CYP11A1 and STAR, decreases. This phenomenon has been observed in many studies [16,19], although there are also reports of both increases [46] and decreases in P4 levels [23]. In Yan’s report [41], it was related that serum levels of P4 and E2 had decreased in the heat-stressed group compared with the control group, but the concentration of P4 in granulosa cells remained unchanged. This may be due to the decrease in total progesterone production caused by a decrease in the number of rhubarb follicles and grade follicles, which are the main producers of progesterone, as well as the decrease in ovarian blood supply and inhibition of neoangiogenesis in ovarian tissue as a result of heat stress. It may also be due to reduced blood supply to the ovary and inhibition of new blood vessel production in the ovarian tissue as a result of heat stress, resulting in reduced hormone flow to the follicles [47]. One study showed that blood progesterone levels in cattle were elevated under high-temperature conditions and speculated that this could be due to progesterone produced by the adrenal glands [6,47]. In contrast, in pigs, an increase in StAR mRNA abundance was observed under the influence of long-term HS [26,48]; trends and specific causes of changes in granulosa cell progesterone levels during heat stress remain to be investigated.

##### In Rodents

Follicle-stimulating hormone (FSH) promotes the production of cAMP, and progesterone enhances the responsiveness of rat GCs to FSH, which further promotes the production of cAMP. It is generally believed that the increase in cAMP is one of the causes of the anti-apoptotic effect of gonadotropins, as it can activate protein kinase A (PKA). However, Peluso et al. demonstrated that the anti-apoptotic effect of P4 does not involve a PKA-dependent mechanism but is mediated by a PKG-dependent mechanism [49]. They also believed that a 14-3-3s protein plays a central role in regulating apoptosis in granulosa cells, possibly by preventing Bax from activating Bax by Bad or by preventing the apoptotic promotion of Bax through its binding to Bax [33,50]. It has also been reported that P4 inhibits mitosis in insulin-dependent small GCs and prevents apoptosis in large GCs [34,51], or blocks the rearrangement and increase of calcium ions to stop apoptosis [35].

However, in mouse GCs, the level of P4 and changes in the expression levels of key genes encoded are not overly complicated, and it has been reported that the degree of reduction in P4 levels in heat-stressed GCs increased significantly with treatment temperature, and that the expression of CYP11A1 and StAR also decreased significantly [29]. Thus, it can be suggested that heat stress may disrupt P4 synthesis by interfering with cholesterol transport and processing. In addition, heat stress can induce autophagic flux and affect progesterone synthesis capacity in mice by reducing P62 levels, increasing LC3B lipidation and binding, and increasing Atg7 expression [36].

#### 2.2.2. In Poultry

In birds, as in mammals in general, CYP11A1, 3β-HSD and StAR remained the principal rate-limiting proteins in the conversion of cholesterol to progesterone; however, the trends observed differed following exposure to heat stress. The mRNA expression levels of StAR, 3β-HSD and CYP11A1 were found to be elevated at the early stages of chicken GC development following in vitro heat stress treatment, and subsequently decreased after 36 h [41]. The expression of StAR and 3β-HSD was similarly elevated in duck GCs, also tested in vitro, but the expression level of CYP11A1 showed a general downregulation trend as in mammals [40]. This difference may be related to the different effects of heat stress in different cellular environments and to the experimental observation that the expression of CYP17A1, the gene encoding P450c17, which is responsible for the conversion of progesterone to androgen, is also reduced after exposure to heat stress [41], probably exacerbating the accumulation of P4.

### 2.3. On Follicle-Stimulating Hormone (FSH)

In both birds and mammals, heat stress inhibits the expression of FSHR in granulosa cells. Experiments in birds have found that activin-A plays a greater role in regulating FSHR expression and granulosa cell sensitivity to gonadotropins. In mammals, more research has focused on the regulation of granulosa cells by FSH and IGF-1 and on anti-apoptotic pathways.

#### 2.3.1. In Mammals

In rodents, peroxides have been shown to significantly inhibit FSH-related cAMP accumulation, and this inhibitory effect persists. However, studies in artiodactyls such as pigs have shown that IGF-1 and FSH can partially relieve this inhibition through different pathways.

##### In Artiodactyla

It has been found that Inhibin regulates follicle-stimulating hormone (FSH) levels in the anterior pituitary gland. In a normal fertile female cycle, there is typically a negative correlation between intra-ovarian steroid hormones (E2 and P4) and FSH. However, in most cases of infertility, this relationship is reversed. This reversal is often attributed to the high production of Inhibin and low production of FSH, which may also exhibit an inverse relationship. Additionally, there is a strong positive correlation between FSH and GSH levels [52]. Inhibition of FSHR expression by HS has been demonstrated experimentally in pigs [24,25] and sheep [27]. This inhibition may be due to the reduced inhibition of negative feedback in smaller follicles stimulated by heat stress [27]. This leads to an increase in the concentration of FSH [32,53] and an over-utilization of these receptors, but has not been reported in cattle. In pigs, heat stress unregulated BimEL Thr112 phosphorylation levels via the JNK pathway to induce apoptosis, and pro-survival factors such as FSH and IGF-1 unregulated BimEL Ser65 phosphorylation levels to inhibit apoptosis in GCs [28].

##### In Rodents

Peroxides were found to significantly inhibit FSH-related cAMP accumulation in rat granulosa cells at low micromolar concentrations, depleting cellular ATP levels within one minute. Prolonged peroxide treatment (60 min) completely blocked the effects of FSH. Interestingly, even after ATP levels returned to normal, the peroxide-induced inhibition of FSH persisted, suggesting that the inhibitory effect of peroxides on FSH is not directly caused by ATP depletion. This suggests that the inhibitory effect of peroxides on FSH is not due to ATP depletion [37], and the detailed mechanism remains to be explored. It has also been found that HS only reduces the number of FSH receptors on the granulosa cells of the antral follicle, but injection of pregnant maternal serum gonadotropin (PMSG) has been found to strongly inhibit FSHR levels and aromatase activity in the granulosa cells, and has shown that FSH-R expression in GCs is also regulated by an E2-dependent mechanism [2].

#### 2.3.2. In Poultry

Activin-A has been demonstrated to stimulate FSHR expression and to play an important role in regulating gonadotropin sensitivity in granulosa cells [42]. The findings revealed that heat stress resulted in a reduction in FSHR expression in laying hens, as evidenced by transcriptomic analysis. Additionally, the study demonstrated that activin-A levels were relatively low, and that activin-B had a minimal impact on LHR and FSHR expression in avian GCs [41].

Reduced FSHR expression levels were also observed in quail and significantly higher levels of 17β-HSD expression were found in the adrenal glands without changes in the ovaries, suggesting that the effect of heat stress on the quail ovary may be mediated through adrenal function [43].

## 3. Regulatory Mechanisms of Apoptosis in Granulosa Cells Due to Heat Stress

### 3.1. Cell Proliferation and Cycle Transition under Heat Stress Invasion

In a high-temperature environment, thermal stress can damage the mitochondrial electron transport chain, leading to electron leakage and the binding of oxygen molecules to form superoxide anions. In addition, ER stress can induce the unfolded protein response (UPR) and increase redox reactions in the cell. The function of the cell’s antioxidant system is also inhibited [54]. The combined effect of these mechanisms leads to an increase in intracellular ROS levels.

#### 3.1.1. In Mammals

In both artiodactyls and rodents, heat stress can cause the ovarian granulosa cells to enter a state of cycle arrest and inhibit their proliferation. This is manifested by a reduction in the expression of PCNA and cyclin. However, studies in rodents and artiodactyls have found that FSH can alleviate this phenomenon in some way.

##### In Artiodactyla

In high-temperature environments, the proliferative ability of ovarian cells with a normal cell cycle is affected, and researchers usually use cell cycle protein B1 and proliferating cell nuclear antigen (PCNA) as markers to study the adverse effects on cells. It was reported that HS significantly reduced the expression of PCNA and cell cycle protein B1 in GCs when porcine GCs were cultured in heat shock in vitro, and it was found that the addition of FSH alleviated the inhibitory effect of HS on PCNA expression [24]. Similarly, a reduction in PCNA expression was observed when bovine GCs were cultured in vitro and treated with HS [8]. Cell cycle arrest occurred in the GO/G1 and G2/M phases [55] and cell proliferation was reduced. Similarly, a study reported that the colony formation rate (CFE) of sheep GCs measured at high temperature was significantly reduced, suggesting that cell proliferation was inhibited by HS [56].

##### In Rodents

Kayampilly et al. showed that FSH increases the mRNA expression of cell cycle protein D2; it also phosphorylates AMPK at ser481/495 via an Akt-dependent pathway and inhibits its activation by decreasing thre172 phosphorylation and reduces the expression of a cell cycle inhibitor (p27kip) to promote granulosa cell proliferation [57]. In mouse liver cells, low-temperature heat stress (e.g., 40 and 42 °C) significantly induced PCNA coding, whereas high-temperature heat stress (e.g., 44 and 46 °C) significantly inhibited PCNA expression [58]. Combined with the previous section “HS decreases FSH levels”, it is reasonable to assume that heat stress indirectly arrests the cell cycle of GCs and inhibits their proliferation by decreasing FSH levels.

#### 3.1.2. In Poultry

According to Yang et al. [40], heat stress inhibits the expression of proliferation genes in duck ovarian granulosa cells, increases the proportion of duck ovarian granulosa cells blocked in the G1 phase, inhibits the expression of proliferation genes required for the transition from the G1 phase to the S phase, and ultimately causes the cell cycle to stagnate in the G0-G1 phase [40], reducing cell proliferation.

### 3.2. Mitochondria Damaged by Heat Stress: Kinetic Imbalance and Autophagy

#### 3.2.1. In Mammals

It is widely acknowledged that cells generate ROS throughout their lifespan, from the moment of their formation until apoptosis. The prevailing view is that ROS are a significant contributor to the damage observed at the molecular level, as well as to the process of aging itself. Furthermore, it is thought that mitochondrial proteins, lipids, and DNA, which are major sources of ROS production, are the primary targets of oxidative stress (Table 2). The functional and structural integrity of mitochondria is maintained by a number of mechanisms, including the interaction of free radical scavengers and antioxidants, repair mechanisms, and the activities of SOD and catalase. Mitochondrial homeostasis is essential for cell survival, and excessive ROS act as signaling molecules to mediate cascade reactions, leading to damage to mitochondrial structure and function [59]. Since ROS levels increase significantly in animals at high temperatures, it is believed that heat stress impairs mitochondrial function and even leads to granulosa cell apoptosis by increasing ROS levels in female ovarian cells, leading to a breakdown in mitochondrial function.

##### In Primates

The critical role of mitochondria in energy production has long been recognized, particularly their ability to protect cells from excessive calcium influx, oxidative damage, and mitochondrial DNA (mtDNA) mutations—key features associated with aging and neurodegenerative diseases. It is well established that oxidative stress can damage mitochondria in human endothelial cells (HUVECs) in vitro, with the extent of damage varying by dose. A twofold increase in reactive oxygen species (ROS) causes transient mitochondrial damage, while a sixfold increase results in not only temporary effects but also permanent impairment of cell proliferation. A twofold increase in ROS transiently damages mitochondria, while a sixfold increase not only has temporary effects but also permanently impairs cell proliferation. This all leads to mitochondrial fragmentation, i.e., increased ROS levels due to oxidative stress can induce mitochondrial fragmentation [77].

##### In Artiodactyla

When cattle are exposed to elevated temperatures, the elevated levels of reactive oxygen species (ROS) in the mitochondria and cytoplasm of oocytes result in the disruption of the genetic material of the cells, leading to cellular dysfunction and, in some cases, permanent damage to organelles, including mitochondria. In addition, it induces the onset of oxidative stress and reduces the mitochondrial energy source and ATP availability, demonstrating that a high temperature impairs mitochondrial autophagy processes and the intracellular accumulation of damaged mitochondria [60]. Similar studies have shown that the expression of Bcl, Bax, caspases, and other cysteine peptidases associated with mitochondrial damage and apoptosis are significantly upregulated in bovine mammary epithelial cells under heat stress and also induce mitochondrial fragmentation by mediating a decrease in the expression of Mfn1/2 and Opal and an upregulation of the expression of Drp1 and Fis-1 [19,61], leading to cytochrome c release.

#### 3.2.2. In Poultry

There are fewer relevant studies in avian animals, but the phenomena observed in these experiments are basically consistent with those observed in animal experiments in the above species. Under the conditions of acute heat stress at 39 °C, ovarian tissue damage in mallard ducks increased the level of apoptosis in GCs, decreased the level of expression of the mitochondrial fusion proteins Mfn1/2 and Opal, increased the level of expression of the mitochondrial fission protein Drp1, and increased the level of autophagy in ovarian mitochondria. Interference with Drp1 and PINK1 has been shown to effectively inhibit acute heat stress-induced mitochondrial autophagy. This inhibition led to decreased levels of LC3II/III and PINK1 proteins, along with increased levels of apoptotic proteins such as Bax. Specifically, interfering with PINK1 significantly reduced apoptosis, whereas interfering with Drp1 did not produce a notable change. These findings suggest that mitochondrial autophagy in heat-stressed mitochondria is primarily mediated through the PINK1 pathway [7].

### 3.3. Heat Stress Affects the Expression of the Heat Shock Protein Family in HSPs

The HSP70 family has unique functions in ovarian granulosa cells. The family is subdivided into inducible HSP70 and structural HSP70, which are located in the mitochondria and endoplasmic reticulum, respectively, and are essential for the correct folding of intracellular proteins and the repair of stress-damaged proteins. Under normal conditions, HSP70 acts as an ATP-dependent molecular chaperone to maintain the steady state of proteins. However, under conditions of heat stress, ovarian granulosa cells rapidly increase the expression of HSP70, which prevents the translocation of Bax proteins to the mitochondria by binding to HSP40, thereby preventing the permeabilization of the mitochondrial outer membrane and inhibiting the release of apoptotic signaling molecules such as cytochrome c, thus effectively inhibiting apoptosis [9].

Expression of HSP genes in the GCs of mice treated with either acute or chronic heat stress increases rapidly, with co-expression of HSP40, HSP70, and HSP90 family members, but chronic heat stress has less effect on ovarian indices, but reduces total body and ovarian growth compared to acute heat stress [63]. Mitochondrial ROS production was induced when HSP60 was reduced in mouse cardiomyocytes under heat stress and was also significantly increased when cytoplasmic Bax was translocated to the mitochondria. Heat treatment of mouse GCs for 6–24 h resulted in a significant increase in the protein level of HSP70, but no significant difference was found when compared with the 48 h group [78].

Following the in vitro culturing of porcine GCs, it was observed that the addition of IGF-1, leptin, and FSH resulted in a reduction in the expression of HSP70.2, HSP72, and HSP105/110 mRNA. Conversely, high-temperature treatment was found to stimulate an increase in the mRNA of the three HSPs. A combination of experiments demonstrated that the addition of IGF-1, leptin, and FSH prevented the stimulatory effect of high temperature on the transcription of HSP70.2, HSP72, and HSP105/110 transcripts. This indicates that the combination of factors prevented stress-related changes in HSPs. Conversely, it was observed that high temperatures reduced the accumulation of HSP70 in granulosa cells [9]. In dairy cow GCs, HSP70 protein accumulation was increased at 24 and 48 h after heat stress treatment, but there was no significant difference in levels between the two and no significant change in mRNA levels, whereas HSP90 expression was significantly upregulated at 24 h after treatment [8]. Bovine GCs from smaller follicles, cultured and exposed to 42 °C, were observed to release an increased number of extracellular vesicles (EVs) enriched in HSP90 and SOD1. Higher expression levels of HSP70 and GRP94 were also detected. Additionally, when cultured GCs were supplemented with stress-associated EVs, they exhibited cellular adaptations in response to subsequent heat stress. However, these adaptations were not evident at 37 °C, indicating that the stress-induced EVs play a role in cellular responses specifically under heat stress conditions [64]. Arabian camel granulosa cells were exposed to 45 °C for two and twenty hours for acute and chronic heat stress treatments, respectively. Acute heat stress induced the expression of HSPA4 and HSPA1B proteins of the HSP70 family as well as DNA damage-binding protein 1, whereas chronic heat stress significantly increased the levels of HSP70A1B, HSP90, and HSP90B1, but a significant increase in HSP70 protein levels was found during recovery, which may reveal the mechanism of camels’ unique recovery and resistance under high-temperature conditions [65].

### 3.4. Heat Stress Triggers the Fas/FasL Extrinsic Apoptotic Pathway

Fas(CD95) is a member of the tumor necrosis factor receptor (TNFR) family. It can directly induce apoptosis of granulocytes by binding to the specific ligand Fas ligand (FasL). This process is particularly critical during heat stress, as high temperatures can directly or indirectly activate the FAS receptor, thereby initiating the apoptosis program. Upon binding to the FAS receptor, FasL facilitates the aggregation of the receptor on the surface of ovarian granulosa cells, concurrently recruiting the adaptor protein FADD (Fas-Associated protein with Death Domain) to the intracellular death domain. This aggregation further recruits caspase-8 via the death effector domain of FADD to form the death-inducing signaling complex (DISC). The activation of caspase-8 within this complex then activates downstream effector caspases, such as caspase-3, which ultimately results in the apoptosis of granulosa cells [10,45,79,80].

Gonadotropins can play a central role as a survival factor in the regulation of Fas/FasL and p53 expression in rat GCs [81], and apoptosis in mouse intestinal epithelial cells was also associated with ROS-induced expression of Fas and FasL [66]. Similarly, Fas and p53 were found to be involved in apoptosis in mouse testicular germ cells exposed to high temperatures [67]; the researchers observed that in porcine granulosa cells, FasL mRNA and protein levels were elevated in GCs with atretic follicles. Furthermore, they noted that both FasL and Fas mRNA and protein levels were increased within these cells compared to GCs with early atresia. This suggests that the Fas/FasL system plays a critical role in the regulation of GC apoptosis [10]. Another study claimed that when chickens were subjected to heat stress, there was an increase in the expression of FasL and Fas in follicular cells, activation of the Fas/FasL system, an increase in apoptosis, and a decrease in the number of follicles, leading to a decrease in egg production rate [45].

### 3.5. Heat Stress Causes Imbalance in Mitochondrial Dynamics

The majority of ROS produced in substantial quantities under conditions of elevated temperature are generated within the mitochondria. Furthermore, it has been demonstrated that the impact of oxidative stress on these organelles is dependent on the dosage [77]. Opening of the mitochondrial permeability transition pore (MPTP) has been shown to be inextricably linked to apoptosis. It has been reported that mitochondrial membrane potential is lost in rat cardiomyocytes under heat stress, and a significant increase in Hsp60 has been found to accompany the translocation of cytoplasmic Bax to the mitochondria [78]. It has also been reported that short-term heat stress increased the expression of HSP72 in porcine oocytes, which induced the phosphorylation of SIRT1 and AMP-activated protein kinase, leading to mitochondrial degradation and the generation of ROS [62]. Mitochondria continuously adapt to the changing cellular environment by fission and fusion. Studies have confirmed that under heat stress, the levels of the mitochondrial fusion proteins mitofusin1/2 (Mfn1/2) and optic atrophy protein 1 (OPA1) decrease in duck ovarian granulosa cells, while the levels of the mitochondrial fission protein Drp1 increase, leading to an increase in mitophagy. It was also found that interfering with Drp1 or PTEN-induced kinase 1 (PINK1) can effectively inhibit mitophagy induced by acute heat stress. However, interfering with Drp1 does not significantly affect the level of apoptosis, whereas interfering with PINK1 leads to an increase in apoptosis levels [7]. A similar phenomenon was confirmed in the mammary epithelium of dairy cows, i.e., Mfn1/2 and Opa-1 were downregulated [61]. Based on the above phenomena, it can be concluded that the occurrence of heat stress disrupts the balance between mitochondrial division and fusion, leading to the release of pro-apoptotic factors such as cytochrome C into the cytoplasm and causing mitochondrial fragmentation. Activation of caspase-9, activation of caspase-3, and the execution of other caspases [82], leading to apoptotic cell death.

### 3.6. Heat Stress Triggers ER Endoplasmic Reticulum Stress

Endoplasmic reticulum (ER) stress occurs when cells are exposed to heat stress, when the balance of the ER is disrupted, leading to the accumulation of folded or misfolded proteins in the ER and to the unfolded protein response (UPR) [83]. Glucose-regulated protein 78 (GRP78) and glucose-regulated protein 94 (GRP94) are often considered as markers and key regulators of ER stress. The mRNA expression of GRP78 and GRP94 was significantly increased in bovine GCs after 24 h of treatment under heat stress conditions and significant accumulation of GRP78 protein was observed, in addition to which the study demonstrated that bovine GCs could respond rapidly to heat stress by activating the expression of GRP94 [8]. Similarly, hyperthermia upregulates GRP78 and GRP94 gene expression in human osteosarcoma cells and is associated with changes in cell membrane calcium levels and increases in calpain expression and activity [68], whereas transcriptional upregulation of GRP78 in GCs during goat follicular atresia suggests that it is more likely to be a major pathway regulating ER stress [69].

The heat stress treatment of mouse granulosa cells (GCs) revealed the activation of the endoplasmic reticulum (ER) stress markers glucose-regulated protein 78 (GRP78) and CCAAT/enhancer binding protein (C/EBP) homologous protein (CHOP). Furthermore, both selenium and boron were found to be effective in inhibiting the ER stress pathway, thereby alleviating heat stress (HS)-induced apoptosis [70,71].

### 3.7. Heat Stress Activates the Nrf2/HO-1 Pathway

In response to heat stress, the excessive production of reactive oxygen species (ROS) triggers cellular defense mechanisms, of which the Nrf2/Keap1 pathway is one of the most important. In unstressed conditions, Nrf2 maintains a basal level in the cytosol through the action of its inhibitory protein Keap1. Upon stimulation, Keap1 dissociates and translocates to the nucleus, forming a heterodimer that binds to the antioxidant response element (ARE). This enables the regulation of GSH production, the activation of the antioxidant genome, and the regulation of key protein expression, thereby maintaining intracellular redox homeostasis [84,85]. In contrast, in dairy GCs, the study by Alemu et al. reported that Nrf2 mRNA expression was increased after 24 h of HS treatment and returned to normal after 48 h [8]. In contrast, Khadrawy et al. found that Nrf2-associated gene expression was also increased in GCs under oxidative stimulation conditions and demonstrated that oxidative stress inhibits the expression of miRNAs targeting bovine Nrf2 (miR-28, 153, and miR-708), while activating Nrf2 expression as a defense mechanism, and that overexpression of these miRNAs may lead to downregulation of Nrf2 expression [72]. The authors suggest that the decrease in Nrf2 mRNA expression after 24 h may be related to the fact that HS inhibits the expression of miRNAs targeting Nrf2, but this has not yet been clearly supported by studies.

In addition, another key defense mechanism regulated by Nrf2 is haem oxygenase-1 (HO-1), an antioxidant enzyme that binds to NADPH and cytochrome P450. It has been reported that in bovine GCs, HO-1 expression increases significantly under heat stress, the silencing of HO-1 increases apoptosis, overexpression inhibits apoptosis by regulating Bax/Bcl-2 expression and cleaved caspase-3 levels, and its metabolite carbon monoxide CO has also been found to increase HO-1 levels and act synergistically with HO-1 to exert a cellular protective effect [73]. A follow-up study by Wang et al. showed that transient heat stress increased the expression of Nrf2 and HO-1 in primary GCs and that HO-1 reduced ROS production and attenuated heat stress-induced apoptosis in bovine GCs [74].

HO-1 mediates harem degradation via bilirubin reductase (BVR) to produce equimolar bilirubin, CO, and Fe^2+^, of which bilirubin is a potent antioxidant. While iron ions are known to promote ROS production via the Fenton reaction, experiments by Balla et al. showed that the onset of ferritin induction in endothelial cells under oxidative stress induced by H_2_O_2_ coincides with the induction of HO-1 [75]. Ferritin is a protective enzyme that acts by sequestering iron ions, and Cheng et al. demonstrated that the synergistic action of the ferritin heavy chain and HO-1 is one of the key reasons for the potent cytoprotective capacity of HO-1 [76]. In addition, CO is one of the products, and Hajime et al. demonstrated its protective effects through the p38MAPK pathway and activation of Akt at low doses, and it was also found that increased levels of CO could in turn increase the levels of HO-1 protein, which could play an anti-apoptotic role by decreasing the phosphorylation of ERK1/2 and inhibiting the activation of the pathway [86,87,88,89,90,91], further enhancing the anti-apoptotic capacity of cells. Taken together, heat stress-induced ROS activate Nrf2 and its downstream HO-1, and the synergistic effect of these two agents constitutes a powerful cellular defense system against oxidative stress.

## 4. Interaction between Heat Stress and Ferroptosis in Granulosa Cells

### 4.1. Heat Stress and Ferroptosis

Ferroptosis is a new form of controlled cell death that mainly depends on the abnormal production and clearance of intracellular reactive oxygen species (ROS) caused by intracellular iron. This leads to a disturbance of the redox balance that mediates lipid-dependent oxidative damage. Iron toxicity is closely related to iron metabolism, amino acid metabolism, and lipid metabolism. The distinguishing features are lipid peroxidation, excessive ROS production, and iron accumulation. However, cells can inhibit these processes through antioxidant systems such as glutathione peroxidase 4 (GPX4) and glutathione (GSH) [92].

GPX4 can inhibit the ferroptosis response and promote cell survival by reducing lipid peroxides to the corresponding non-toxic lipid alcohols, i.e., factors that induce ferroptosis can directly or indirectly inhibit GPX4 levels and induce oxidative cell death via the Fenton reaction (Fenton). In addition to this, elevated levels of lipid peroxides have been found to induce ferroptosis in tumor cells [93]. Correspondingly, it has been found that the expression of GPX4 and ferritin is suppressed under heat stress, and the protein level of intracellular transferrin receptor 1 (TFR1) is increased, promoting the accumulation of lipid peroxides in porcine Sertoli cells [94]. There is no doubt that the massive production of ROS in excess of the level of GPX4 antioxidant activity under heat stress conditions may in part drive the onset of ferroptosis.

### 4.2. Ferroptosis and Granulocytes

Ferroptosis, a form of cell death, is mainly characterized by the reduction in or disappearance of mitochondrial cristae, rupture of the outer mitochondrial membrane, and condensation of the mitochondrial membrane. The mitochondrial autophagy pathway can degrade ferritin, and ferroptosis regulatory proteins are also involved in regulating autophagy, and it has been shown that excessive autophagy can also promote ferroptosis through iron accumulation or lipid peroxidation [95].

Mitochondrial metabolism and ferritin deposition are closely related; the study by Zheng et al. found that both ferroptosis and apoptosis were present at the onset of the Fenton reaction, suggesting that apoptosis can also induce the onset of ferroptosis [96]. In porcine early atretic follicles, the expression of transferrin (TF) was significantly reduced, the expression of iron chaperone protein (PCBP) was significantly increased, and glutathione metabolism was also abnormal, suggesting that iron accumulation had occurred in granulosa cells at this stage of the follicle. Combined with the significant increase in the expression of marker genes closely related to granulosa cell apoptosis, such as FASLG, CASP3, and BAK1, reported in the study, it can be concluded that ferroptosis induced the development of apoptosis in granulosa cells at this stage of the atretic follicle. The study reported a significant increase in the expression of marker genes closely related to granulosa cell apoptosis, such as FASLG, CASP3, and BAK1. This suggests that ferroptosis plays a role in inducing apoptosis in granulosa cells during the early stages of follicular atresia. The upregulation of these apoptosis-related genes supports the conclusion that ferroptosis contributes to the initiation of apoptosis in granulosa cells at this stage of follicle development [97].

### 4.3. Interaction Mechanism

**Figure 1:**
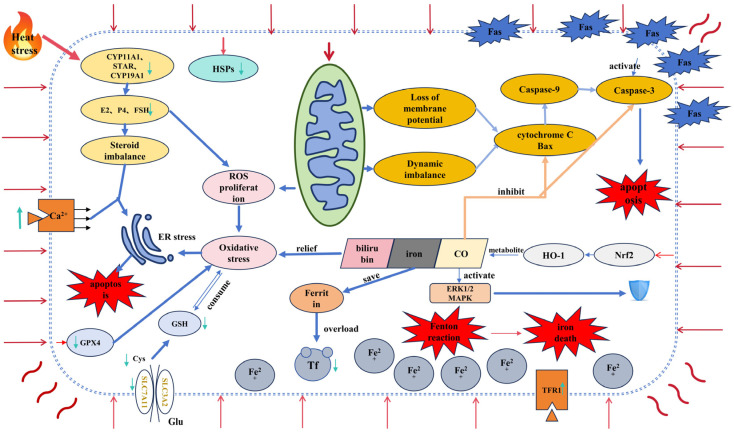
Heat stress increases intracellular calcium influx and ROS production in granulosa cells, disrupts steroid homeostasis, decreases the expression of GPX4 and SLC7A11, which is importantly associated with GSH levels, increases intracellular oxidative stress, and induces the onset of ER stress. Loss of mitochondrial membrane potential and kinetic imbalance, release of cytochrome C-induced activation of caspases, and a series of apoptotic factors, in concert with the FAS/FASL pathway, triggered apoptosis. Metabolism of iron ions from the protective Nrf2/HO-1 pathway induces ferroptosis when the expression level exceeds the tolerance range of ferritin, whose expression is inhibited by heat stress. Red line with arrows or red wavy line: high-temperature environment stresses granulosa cells and brings about effects; dark blue line with arrows: leads to downstream mechanisms; light blue line with arrows: release or activation; green line with arrows: change in level; black line with arrows: level of entry into the cell; orange line with arrows: inhibition of release or activation; blue shield: exerts a protective effect on granulosa cells.

Taken together with the previous description of the levels of haem oxygenase (HO-1), we can see that it has a dual role in the regulation of granulocyte death. When heat stress activates the defense mechanism of the Nrf2/HO-1 pathway, there is no doubt that HO-1 plays a protective role, and not only does it degrade heme to Fe^2+^, but its metabolites also play an anti-apoptotic role within the granulocyte. However, many recent studies have shown that although HO-1 expression is controlled by the Nrf2/antioxidant response signaling pathway, in parallel with the activation of the cellular antioxidant mechanism, HO-1 plays a role in the detoxification of hemoglobin to biliverdin, a process that leads to the release of labile iron, which is often implicated in oxidative stress-mediated cell death [98]. More importantly, the upregulation of HO-1 in cells, including GCs, can lead to several changes, such as elevated iron ion levels, decreased GPX4 expression, and increased lipid peroxidation. These alterations promote ferroptosis, contributing to cellular toxicity [11,98,99]. Additionally, mitochondria-targeting HO-1 has been shown to reduce cytochrome C oxidase activity, which in turn increases ROS production. This oxidative stress leads to elevated levels of the mitochondrial division protein Drp1 and the autophagy marker LC-3, further exacerbating mitochondrial damage [100,101]. Suttner and Dennery et al. clearly demonstrated that low levels (less than 5-fold) of HO-1 expression were cytoprotective and high levels (greater than 15-fold) of HO-1 expression were associated with significant oxygen cytotoxicity [99]. In addition, it was found that interference with Nrf2 in bovine mammary epithelial cells resulted in a decrease in intracellular protein expression of GPX4, SLC7A11, and ferritin heavy chain 1 (FTH1), suggesting that Nrf2 is an important component of cellular defense against the onset of ferroptosis [102]. Therefore, we propose that a ferritin tolerance threshold exists when heat stress activates the Nrf2/HO-1 defense mechanism in animal ovarian granulosa cells. The expression levels of HO-1 and ferritin may determine whether the Nrf2/HO-1 pathway promotes or inhibits the onset of cellular ferroptosis (Figure 1). Specifically, heat stress could lead to an abnormal accumulation of iron ions by disrupting the balance between HO-1 and ferritin expression. Further investigation is needed to elucidate the precise mechanisms involved (Table 3).

## 5. Conclusions and Prospects

By ‘cross-pathway’, we mean the interaction and regulation between multiple cell death signal pathways triggered by heat stress. ROS production, the Nrf2/HO-1 signal pathway, and mitochondrial damage under heat stress are all mechanisms involved in both apoptosis and ferroptosis. Therefore, we believe that these two forms of cell death are not completely independent but influence each other through shared upstream signals and molecular mechanisms. Most importantly, the regulation of the Nrf2/HO-1 pathway not only affects antioxidant capacity but may also promote ferroptosis when over-activated. Therefore, these common mechanisms may form a ‘cross-pathway’. That is, the two forms of cell death interact with each other through cross-regulation. This cross-regulation is crucial because it allows for greater flexibility in the cellular response, enabling the cell to dynamically adapt to heat stress by simultaneously modulating both apoptosis and ferroptosis. This flexibility optimizes the stress response by ensuring efficient removal of damaged cells, preserving ovarian function, and preventing further tissue damage. A multi-faceted approach targeting cross-talk pathways may be considered to mitigate the adverse effects of heat stress on ovarian granulosa cells, particularly apoptosis and ferroptosis. Antioxidant therapies, such as the use of glutathione, vitamin E, and selenium, are effective in neutralizing excess ROS and inhibiting the activation of cell death pathways. In addition, iron chelation therapy can reduce iron accumulation and lipid peroxidation, thereby significantly reducing ferroptosis. Enhancement of the activity of heat shock proteins (e.g., HSP70 and HSP90) could also enhance the cellular defense against thermal injury. Enhancement of heat shock proteins (e.g., HSP70 and HSP90) also enhanced the cellular defense against thermal injury. Modulation of the Nrf2/HO-1 pathway is essential for antioxidant protection, but the risk of promoting ferroptosis by over-activation needs to be avoided. These strategies may provide a theoretical basis for the protection of ovarian function and enhancement of the adaptation of the livestock organism to environmental stress.

Future studies should further explore the detailed mechanisms by which heat stress affects ovarian tissue structure and function at the molecular level, in particular mitochondrial autophagy and the interaction between heat stress and ferroptosis under heat stress conditions. In addition, further studies on the mechanisms of heat stress-induced ferroptosis are needed to explore potential protective strategies, such as antioxidant applications, interventions to improve iron metabolism, and the development of novel anti-apoptotic therapies. At the same time, research should focus on how to attenuate or prevent heat stress-induced cellular damage by modulating these pathways.

In terms of technology and methodology, the adoption of high-throughput genomics, proteomics, and metabolomics approaches could provide a more comprehensive perspective for understanding the complex network of heat stress effects on ovarian function. Additionally, the development and utilization of in vitro model systems, such as organ cultures and cell lines, for simulating heat stress will facilitate the study of its effects on ovarian tissue within a controlled environment. These models will provide crucial experimental tools for research. Understanding the mechanisms by which heat stress influences ovarian tissue and triggers ferroptosis will offer a theoretical foundation for developing new therapeutic strategies. Such advancements could potentially improve or maintain ovarian health and reproductive function in the future.

## Figures and Tables

**Table 1:** Heat stress affects steroid hormone production.

Taxonomic Group	Types/Strains	Heat Stress Environmental Settings	Main Findings	References
1. Bovine	Dairy cattle	36 °C and relative humidity 60% for five days	• Heat stress reduces E2 levels in GCs Heat stress has a delayed effect on steroid hormone secretion	[14]
	Bovine	/	• FSH synergizes with IGF1 to increase cell number and CYP19A1 mRNA expression in bovine granulosa cells	[15]
	Dairy cattle	38 °C or 40.5 °C for 12 h,	• Heat stress induced GC apoptosis through the BAX/BCL-2 pathway Heat stress reduced the steroidogenic gene messenger RNA (mRNA) expression and E2 synthesis	[16]
	Dairy cattle	(28 °C, 52% relative humidity, THI = 76) conditions for a total of 95 h	• Acute preovulatory heat stress specifically alters the gene expression profile of granulosa cells but does not induce stress-related genes and pathways	[17]
	Holstein dairy cows	Winter maintained at (36, 60% RH, THI = 88.3) for 3 days, 12 h per day; summer	• Seasonal heat stress has less effect on GC (estradiol-producing) biosynthesis capacity	[18]
	Dairy cattle	(39, 40 and 41 °C) for 2 h	• HS significantly reduced E2 and P4 levels in bGCs, while increasing intracellular ROS and apoptosis rates • HS can significantly regulate many differentially expressed genes (DEGs), such as BCL2L1, STAR, and CYP11A1, which are involved in apoptosis, steroidogenesis, and oxidative stress	[19]
	Egyptian buffalo (Bubalus bubalis)	Heat treatment at 40.5 °C for 24, 48 or 72 h	• Buffalo GCs treated with heat stress for 72 h maintain normal vigor, steroidogenesis, and transcriptional profiles	[20]
	Holstein dairy cattle	(43.3 °C) for seven days, eight hours per day	• Estradiol and progesterone levels in follicular fluid of dominant follicles did not differ after HS treatment	[21]
	Lactating Holstein cows	/	• Acute heat stress has no significant effect on follicular fluid PO2, estradiol-17b, or progesterone concentrations	[22]
	Non-pregnant cows	43 °C for 2 h	• Acute heat stress decreases P4 concentrations in bovine GCs	[23]
	Holstein heifers	Incubate with 0, 2, or 100 ng/mL LH or FSH for 96 h at 37, 39, or 41 °C	• Elevated blood progesterone levels in cattle under high-temperature conditions	[6]
2. Pig	Slovakian white gilts	41.5 °C for 48 h	• Hormones (IGF-I, leptin, and FSH) are involved in the control of proliferation, apoptosis, and secretory activity of ovarian cells • Heat stress leads to reduced ovarian cell proliferation and apoptosis and excessive ovarian hormone secretion	[24]
	Prepubertal gilts	Pre-incubation at 41 °C for 24 h followed by three hours of incubation in a humid environment at (41 °C, 5% CO_2_)	• LPS and heat stress could impair estradiol biosynthesis in GCs via increased HSP70 and indirect inhibition of Smad3 phosphorylation and nuclear translocation	[25]
	Female pigs	(35 °C; 20–35% humidity; *n* = 6) for either 7 (*n* = 10) or 35 d (*n* = 12)	• HS increased mRNA and protein abundance and phosphorylated AKT (pAKT) in ovine CYP19a and STAR	[26]
	Malabari female goats	Exposure to outdoor summer heat stress for 45 days (6 h per day)	• HS inhibits FSHR expression	[27]
	Gilts	/	• Pro-survival factors such as FSH and IGF-1 upregulate the phosphorylation level of BimEL-Ser65 to inhibit apoptosis in GCs	[28]
3. Mice and rats	Kunming mice	CO_2_ incubator at 40 °C or 43 °C	• Heat stress reduces E2 and P4 secretion and mRNA expression of steroid-related genes and induces apoptosis in GCs via the mitochondrial pathway	[29]
	C57BL/6J mouse	/	• E2 enhances Ape1 expression and reduces oxidative DNA damage	[30]
	Wistar female rats	35 °C and 70% relative humidity conditions for 48 h	• Heat stress reduces the number of FSH receptors on the granulosa cells of the antral follicle and the level of estradiol in the follicular fluid, and strongly inhibits gonadotropin receptor levels and aromatase activity in the granulosa cells	[2]
	Female rats	45 °C water bath incubation for 10 min	• Two transcription-dependent responses: heat shock-induced anti-steroid response and HPS induction, which occur simultaneously	[31]
	Sprague Dawley rats	38 ± 0:5 °C, RH55 ± 5% for 2 h/day for at least 90 days	• Heat stress treatment decreased the growth levels of Hsp70 and CORT, decreased the levels of E2 and LH, increased the levels of FSH and Prl, did not differ in GnRH and T4, and significantly decreased INS	[32]
	CF-1 mice	/	• The phosphorylation state of 14-3-3 is regulated by P4 through a PKG-dependent pathway	[33]
	Wistar rats	/	• P4 inhibits insulin-dependent mitosis in small GCs	[34]
	Wistar rats	/	• P4 acts through its receptor to prevent a redistribution and increase in [Ca^2+^] that may subsequently result in GC apoptosis	[35]
	Mice	Seven-day continuous exposure to 40.5 ± 0.2 °C	• Heat exposure decreases serum progesterone levels and ovarian P450scc expression and can induce autophagic flux and affect progesterone synthesis in mice by decreasing P62 levels, enhancing LC3B lipidation and binding, and increasing Atg7 expression.	[36]
	CD rats	/	• The inhibitory effect of peroxides on FSH is not due to ATP depletion	[37]
4. Poultry	60-wk-old Hy-Line laying hens	32 °C (25% RH) for three weeks.	• ROS formation was significantly increased by heat exposure in laying hens	[38]
	Laying hens	37 °C with 5% CO_2_	• SLC5A5 deficiency inhibits proliferation, steroid hormone synthesis, and secretion and promotes apoptosis in chicken GCs	[39]
	Shan Ma ducks	39 °C, 5% CO_2_ for 6 h	• The expression of proliferative genes required for the transition from G1 to S phase is inhibited Heat stress inhibits GC estradiol synthesis by downregulating CYP11A1 and CYP19A1 gene expression	[40]
	Hy-Line laying hens	32 °C, 50%RHh for 14 days	• Heat stress enhanced P4 secretion by increasing the expression of StAR, CYP11A1, and 3β-HSD and inhibited estradiol synthesis by suppressing the expression of FSHR and CYP19A1, and upregulation of HSP70 was also observed	[41]
	Single-comb White Leghorn hens of the Babcock B 300	/	• Activin A significantly increased FSHR expression in follicular granulosa cells	[42]
	Female Japanese quail of a laying strain	34 °C for 4 h per day (12:00 to 16:00) for 10 consecutive days	• The expression level of 17β-HSD under heat stress was unchanged in the ovary, but significantly increased in the adrenal gland (*p* < 0.05) • The effect of heat stress on the ovaries of female quail is mediated by adrenal function	[43]

**Table 2:** Regulatory mechanisms of apoptosis in granulosa cells due to heat stress.

Cellular Regulatory Processes	Types/Strains	Heat Stress Environmental Settings	Main Findings	References
1. Cell proliferation and cycle transition under heat stress invasion	Slovakian white gilts	41.5 °C	• HS significantly reduced the expression of PCNA and cell cycle protein B1 in GCs, and the addition of FSH alleviated this effect	[24]
	Bovine	Heat treatment 24 and 48 h	• Reduction in PCNA expression in GCs was observed after treatment with HS	[8]
	Holstein cattle	2 h at 40 °C in humidified air with 5% carbon dioxide.	• Cell cycle arrest in GO/G1 and G2/M phases	[55]
	Sheep	5% CO_2_ in humidified air at 43 °C for 2 h for 12 days.	• Colony formation rate (CFE) of sheep GCs was significantly reduced under heat stress	[56]
	Rats	/	• FSH promotes granulosa cell proliferation by increasing cell cycle protein D2 mRNA expression and through an Akt-dependent pathway	[57]
	Mouse	40, 42, 44, and 46 °C for 20 min at 60% relative humidity	• Low-temperature heat stress (e.g., 40 and 42 °C) significantly induced PCNA orchestration, whereas high-temperature heat stress (e.g., 44 and 46 °C) significantly inhibited PCNA expression	[58]
	Shan Ma ducks	41 and 43 °C for 24 h	• In ducks, during heat stress, GC is blocked at the G1 phase and the expression of proliferation genes is inhibited	[40]
2. Mitochondria damaged by heat stress: kinetic imbalance and autophagy	Cows	39.8 and 41.0 °C for 6 and 12 h.	• Heat stress impairs mitochondria and their autophagic processes, leading to an accumulation of damaged mitochondria in the cell	[60]
	Dairy cow mammary epithelial cells	42 °C for 2 h	• Mfn1/2 and Opa-1 are downregulated and Drp1 and Fis-1 are upregulated following heat stress	[61]
	Duck	43 °C treatment (0, 60, 90, 120 and 150 min)	• Interfering with PINK1 levels significantly reduced apoptosis levels, but interfering with Drp1 levels did not change significantly	[7]
	Porcine	41.5 °C for 1 h	• Short-term heat stress increases the expression level of HSP72 in porcine oocytes, induces phosphorylation of SIRT1 and AMP-activated protein kinase, and induces mitochondrial degradation and generation	[62]
3. Heat stress affects the expression of the heat shock protein family in HSPs	ZCK mice	43 °C at 50% humidity for 0.5 h; 1.5 h per day (12:00–13:30) at 39 °C with 50% humidity for 1, 3 or 6 weeks.	• Small HSP genes increase rapidly after heat treatment and are co-expressed by HSP40, HSP70, and HSP90 family members	[63]
	Slovakian white gilts	41.5 °C for 2 days	• IGF-1, leptin, and FSH prevent stress-induced changes in HSPs • HS reduces the accumulation of HSP70 in granulosa cells	[9]
	Bovine	Heat treatment 24 and 48 h	• HSP70 protein accumulation increased at 24 and 48 h after heat stress treatment but there was no significant difference in levels between the two and no significant change in mRNA levels • HSP90 expression was significantly upregulated at 24 h after treatment	[8]
	Bovine	42 °C for 24 h	• EVs produced by HS-exposed granulosa cells were enriched in HSP90 and SOD1, and HSP70 and GRP94 were also highly expressed, allowing recipient cells to be robust to subsequent HS	[64]
	Camel	After incubation at 45 °C for 2 h or 20 h, place at 38 °C	• Acute heat stress induces the expression of HSPA4, HSPA1B protein, and DNA damage binding protein 1 • Chronic heat stress significantly upregulates the levels of HSP70A1B, HSP90, and HSP90B1, but HSP70 protein levels significantly increase during recovery	[65]
4. Heat stress triggers the FAS/FASL extrinsic apoptotic pathway	Mature sows	/	• Upregulation of FasL and Fas mRNA and protein levels in granulosa cells during follicular atresia • Fas is transferred from the cytoplasm to the cell membrane region at the same time as the granular cells begin to undergo apoptosis	[10]
	Mode-K cells derived from C3H/HeJ mice	/	• ROS-induced expression of Fas and Fas-L	[66]
	Mice	/	• Fas and p53 are involved in apoptosis in mouse testicular germ cells	[67]
	Laying hens	35–37 °C for 5 days	• HS triggers follicular cell apoptosis by activating the FasL/Fas and TNF-α systems.	[45]
5. Heat stress triggers ER endoplasmic reticulum stress	Bovine	Heat treatment 24 and 48 h	• The mRNA expression of GRP78 and GRP94 was significantly increased in bovine GCs, and a significant accumulation of GRP78 protein was observed • Bovine GCs can respond rapidly to heat stress by activating GRP94 expression	[8]
	Human OS cell line	/	• High temperatures can upregulate the expression of GRP78 and GRP94 genes in cells, accompanied by changes in calcium levels in cell membranes and an increase in the expression and activity of calpain	[68]
	Goat	/	• Transcriptional upregulation of GRP78 in GCs during follicular atresia in goats	[69]
	Mice	Incubate at 40 °C for 24 h in 5% CO_2_	• HS can induce apoptosis of mGCs through the ER stress pathway, and boron can alleviate this to a certain extent	[70]
	Mice	Incubate at 39 °C for 0, 6, 12, 24, and 48 h in 5% CO_2_	• Selenium is protective against chronic HS-induced apoptosis by inhibiting the ER stress pathway	[71]
6. Heat stress activates the Nrf2/HO-1 pathway	Bovine	Heat treatment 24 and 48 h	• Nrf2 mRNA expression was elevated after 24 h of HS treatment and then normalized after 48 h	[8]
	Bovine	/	• Oxidative stimulation activates the expression of Nrf2-associated genes in GCs and represses miRNAs targeting Nrf2, and overexpression of these miRNAs leads to downregulation of Nrf2 expression	[72]
	Bovine	39 and 40 °C for 2 h	• HO-1 expression is significantly increased by heat stress • Silencing of HO-1 increases apoptosis • HO-1 inhibits apoptosis	[73]
	Bovine	40 °C and 42 °C for 2 h	• HO-1 reduces reactive oxygen species production and activates antioxidant responses	[74]
	Porcine aortic endothelial cells	/	• Ferritin induction in endothelial cells under oxidative stress induced by H_2_O_2_ occurs concomitantly with HO-1 induction	[75]
	Mice	/	• The synergistic effect of ferritin heavy chain and HO-1 is one of the key reasons for the potent cytoprotective capacity of HO-1	[76]

**Table 3:** Interaction between heat stress and ferroptosis in granulosa cells.

Research Fields	Types/Strains	Heat Stress Environmental Settings	Main Findings	References
1. Heat stress and ferroptosis	Human	/	• Elevated lipid peroxide levels induce ferroptosis	[93]
	Porcine Sertoli cells	5% CO_2_ incubator at 44 °C for 30 min	• Expression of GPX4 and ferritin is inhibited under heat stress Increased protein levels of intracellular transferrin receptor 1 (TFR1)	[94]
2. Ferroptosis and granulocytes	Pig	/	• Within early atretic follicles, there is a significant decrease in the expression of transferrin, a significant increase in the expression of iron chaperonin, and abnormalities in glutathione metabolism	[97]
3. Interaction mechanism	Human	/	• HO-1 acts to detoxify hemoglobin to biliverdin, a process that leads to the release of labile iron	[98]
	Hamster fibroblast (HA-1) cells	/	• Low levels (<5-fold) of HO-1 expression were cytoprotective, and high levels (>15-fold) of HO-1 expression were associated with significant oxygen cytotoxicity	[99]
	Mice	/	• Iron-overloaded follicular fluid induces granulosa cell iron metamorphosis and releases granulosa cell exosomes containing aberrant miRNAs	[11]
	Mouse	/	• Mitochondrial targeting of HO-1 reduces cytochrome C oxidase activity, induces ROS production, and leads to an increase in mitochondrial fission preprotein Drp1 and autophagy marker LC-3 in mitochondria	[103]
	Hepatocellular carcinoma cells	/	• Activation of the p62-Keap1-NRF2 pathway protects against ferroptosis in hepatocellular carcinoma cells	[100]
	Bovine mammary epithelial cells	/	• Interference with Nrf2 in bovine mammary epithelial cells reveals decreased intracellular protein expression of GPX4, SLC7A11, and ferritin heavy chain 1 (FTH1)	[102]

## Data Availability

The authors confirm that all the data used in the article supporting this study are available within the article.
